# Age- and sex-related differences in myosin heavy chain isoforms and muscle strength, function, and quality: a cross sectional study

**DOI:** 10.20463/jenb.2018.0016

**Published:** 2018-06-30

**Authors:** Seung-Lyul Oh, Sang Hoon Yoon, Jae-Young Lim

**Affiliations:** 1 Aging & Mobility Biophysics Laboratory, Department of Rehabilitation Medicine, Seoul National University Bundang Hospital, Seongnam Republic of Korea; 2 Department of Neurosurgery, The Armed Forces Capital Hospital, Seongnam Republic of Korea; 3 Institute on Aging, Seoul National University, Seoul Republic of Korea

**Keywords:** Myosin heavy chain, Muscle strength, Muscle quality, Sex-related difference, Aging

## Abstract

**[Purpose]:**

Declining muscle strength and function are hallmarks of the aging process. This study aimed to determine sex-related differences in myosin heavy chain (MHC) isoforms and muscle mass, strength, and quality with aging.

**[Methods]:**

This cross-sectional study recruited 53 healthy participants (32 men, 21 women) aged 20–85 years who were divided into four groups: young men (n=17, YM, 29.23±4.51), older men (n=15, OM, 71.87±3.42), young women (n=11, YW, 29.64±4.88), and older women (n=10, OW, 68.1±1.91). Body composition and muscle strength and quality were analyzed. Muscle specimens were obtained from the vastus lateralis in all participants to analyze the type of MHC isoforms.

**[Results]:**

Men showed a greater age-related decline in skeletal muscle mass (18.6%, p<0.01), lean body mass (10.1%, p<0.05), grip strength (35.3%, p<0.001), isometric strength (29.6%, p<0.001), isotonic power (42.5%, p<0.001), isokinetic strength (up to 44.3%, p<0.001), and muscle quality (up to 24.8%, p<0.01). In contrast, women had significantly lower isometric strength (24.2%, p<0.05), isotonic power (36.5%, p<0.01), and upper-body muscle quality (24.7%, p<0.001) with aging. In addition, the proportion of MHC IIa was significantly lower in OM (p<0.05) and OW (p<0.05) than in YM and YW, respectively. However, the proportion of MHC I was significantly higher in OM (p<0.01) than in YM but was high in both YW and OW. MHC I and MHC IIa negatively and positively correlated, respectively, with muscle strength and function.

**[Conclusion]:**

These results indicate the existence of sex-related differences in muscle mass, strength, and quality and MHC isoform composition with increasing age. The effects on muscle strength and function with aging were significant in men, but not in women. Higher and lower proportions of MHC I and MHC IIa fibers, respectively, were inversely associated with muscle strength and quality. In particular, Korean YW showed lower muscle strength and quality, and the proportion of MHC isoforms was similar to that in the muscles of OW.

## INTRODUCTION

Muscle strength normally declines with aging, and its measurement is an important component of physical examination for the evaluation of physical function^1^. Muscle strength reaches its peak at ages between 25 and 35 years and plateaus until the age of >40 years, although it may slightly decrease and starts to quickly decline starting at the age of 60 years. Although the decline rates of muscle strength after the age of 65 years vary for each individual, it has been reported that an average of 4.5–5.5% of muscle strength is lost every 5 years^2^. Therefore, muscle strength decreases by >30% on average by the seventh and eighth decade of life among the elderly^3^.

Maintenance of muscle mass and strength is an important contributing factor to optimal health and physical functional status in older adults, as the loss in muscle mass and strength with increasing aging results in sarcopenia and ultimately increases susceptibility to injury and prevalence of their mobility limitations. Although many previous studies have shown sex-related differences in muscle mass, strength, and quality with aging^4-14^, few have reported sex-related differences in these muscle parameters in relation to sarcopenia in the Korean population.

Skeletal muscle mass index (SMI) (appendicular skeletal muscle mass [ASM]/height squared) and muscle strength in Korean men decrease with aging. In contrast, in Korean women, no significant differences between the young and older groups have been observed, who instead showed a tendency toward an increase with aging^15,16^. Kim et al.^15^ reported sex-related differences in muscle mass and SMI using data from the Korean National Health and Nutrition Examination Survey, which is a nationwide population-based cross-sectional study, from 2008 to 2010. Total muscle mass and ASM in men dramatically increased in the third decade of their life and then gradually decreased until the age of 90 years, with a slight acceleration after the age of 60 years. However, changes in SMI and muscle strength with aging occurred quite differently in women, indicating that total muscle mass and ASM in women increased slowly until the fourth decade of their life, remained constant until the fifth and sixth decades, and subsequently started to decrease. The total amount of increment or decline in muscle mass and strength change with aging was expectedly less in women than in men.

Muscle loss due to aging appears mainly in fast-twitch myosin heavy chain (MHC) fibers^17-19^. The proportion of fast-twitch MHC fibers (type II isoform dominant muscle) positively correlates with muscle strength^18,20^. However, Short et al.^18^ reported that MHC IIa isoform was associated with muscle strength in both men and women, but no significant correlation was shown for MHC IIx isoform. Previous studies have investigated MHC distribution and adaptive changes in response to various interventions such as endurance exercise training^18,21,22^ and resistance exercise with^23,24^ or without bed rest^25^, which led to muscle hypertrophy or atrophy.

Although the change in MHC isoforms with aging is well known, to our knowledge, age- and sex-related differences in muscle strength, function, and quality between MHC isoform distributions have not been well investigated in human skeletal muscles. This cross-sectional study aimed to analyze the proportion of MHC isoforms and the correlation between these isoforms and muscle strength and quality with aging and to determine sex-related differences in muscle mass, strength, and quality with aging in young and old men and women.

## METHODS

### Participants

Participants were recruited from Seoul National University Bundang Hospital (SNUBH) by advertisement and verbal invitation. Participants with (1) age ≥65 years for the elderly and 20–40 years for the adults; (2) no difficulty in independent ambulation; (3) no difficulty in walking for 400 m and climbing 10 steps; and (4) score ≥10 on the Short Physical Performance Battery (SPPB) were included. Further, those with (1) any severe musculoskeletal injuries or problems; (2) any previous bone fracture surgery within 6 months from the test date; and (3) systolic blood pressure ≥150 mmHg or diastolic blood pressure ≥90 mmHg who underwent treatment for or had history of hypertension were excluded from this study. Participants were asked to provide informed consent and were subsequently checked against the inclusion criteria by a clinical specialist. The protocol was approved by the institutional review board of SNUBH (B-1307-212-008).

In total, 53 healthy participants provided informed consent and were included in this study. Participants were divided into two different age groups: young (Y, *n*=28, 20–40 years) and older (O, *n*=25, >65 years). Each group was further divided according to sex: men (M, n=32) and women (W, *n*=21). Finally, the four groups in this study were as follows: young (17 young men, 11 young women) and older (15 older men, SPPB score [M±SD]: 11.6±1.06; 10 older women, SPPB score [M±SD]: 11.6±0.7). Participants’ characteristics are shown in Table 1.

**Table 1.** Participant characteristics

Characteristic	Y (n=28)	O (n=25)	YM (n=17)	OM (n=15)	YW (n=11)	OW (n=10)	Interaction (gender X age)
Age, yr.	29.39±4.57	70.36±3.43	29.23±4.51	71.87±3.42	29.64±4.88	68.1±1.91	478.084***
Height, cm	170.42±8.92	161.42±6.86*	176.94±2.48	165.2±5.09a	160±35±4.57	155.76±5.07b	62.317***
Weight, kg	68.28±13.37	64.54±8.83	75.71±9.04	67.95±5.83a	56.79±10.64	59.44±10.33	12.684***
BMI, kg/m^2^	23.34±3.31	24.78±3.18	24.17±2.67	24.96±2.55	22.06±3.89	24.5±4.08	2.119

The data presented as mean±SD. Y, young adult; O, older adult; YM, young men; OM, older men; YW, young women; OW; older women. The body mass index (BMI) was defined as body weight divided by height squared (body weight/height^2^). *P* values were calculated by independent T-Test. Significantly different between groups (a, YM vs. OM; b, YW vs. OW). Statistical significance was set at p<0.05.

### Experimental procedures

All participants in this study visited the research laboratory thrice. During the first and second visits, the screening procedure for each participant and the measurement of body composition, muscle strength, and muscle quality were performed, respectively. During the third visit, skeletal muscle specimens were acquired through biopsy from all participants for the analysis of MHC isoforms. Participants were restricted to excessive food intake and strenuous physical activity 24 h before each visiting time.

### Body composition

Total and regional lean mass and total fat mass were assessed using dual-energy X-ray absorptiometry (DEXA) (PIXI; GE Medical Systems Lunar, Madison, WI, USA). The legs were defined using a line bisecting the femoral neck. Bone mineral content was subtracted from the total and regional lean mass to calculate the quantity of total non-bone lean mass, which primarily represents the skeletal muscle^26^. Fat mass was estimated for the whole body as well and was examined for these analyses. Moreover, body mass index (weight divided by height squared) in kg/m^2^ was used to measure body composition. SMI was defined as ASM divided by height squared.

### Muscle strength, function, and quality

We measured grip strength using a hand dynamometer (Jamar 5030JI; Bolingbrook, IL, USA) and maximal voluntary isokinetic/isometric strength and isotonic power of the dominant knee extensor and flexor using an isokinetic dynamometer (Primus RS; BTE, Hanover, MD, USA). Grip strength was measured twice on each hand, with the elbow flexed at a right angle and the forearm in neutral position^4^. The maximum of the four readings generated was considered the maximal grip strength. Maximal voluntary concentric isokinetic torque was assessed in Newton-meters (Nm) at angular velocities of 60 deg/s, and it also presented as relative isokinetic strength which is a value divided by body mass (Nm/kg, %). For at least three times but no more than six, maximal efforts were allowed to produce three overlying curves, and peak maximal torque production was recorded. Peak isometric knee extensor torque at 90° of knee flexion was measured following the same procedure. For this test, participants were asked to extend their knee as fast and hard as they could to maintain force production for 3 s. Maximal isotonic power of the muscle was measured in watts (W). Muscle quality was evaluated using the strength-to-muscle mass ratio^27^. We defined the upper- and lower-body muscle qualities as the ratio of the grip strength (in kg) and isokinetic torque (in Nm) at the arm and knee to the lean mass (in kg) in the arm and leg, respectively, as measured using DEXA.

### Muscle biopsy

Muscle specimens were percutaneously obtained under local anesthesia from the vastus lateralis using a modified Bergström needle (11750-06 and 11750-07; Dixons Surgical Instruments, Wickford, UK) biopsy technique with suction. We performed muscle biopsy under ultrasound guidance to minimize vessel and nerve damage. Muscle samples were embedded in O.C.T. compound (Tissue-Tek; CA, USA), frozen with liquid nitrogen-cooled isopentane, and stored at -80°C for electrophoretic determination of MHC isoform composition.

### Myosin heavy chain

**Fig. 1.**
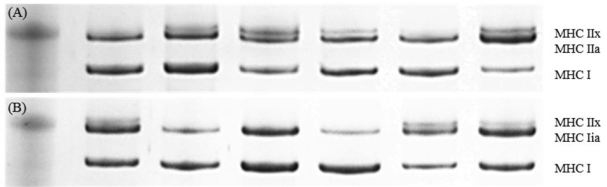
Myosin heavy chain isoforms. Representative electrophoretic separation (SDS-PAGE) of myosin heavy chain isoforms in human skeletal muscle oyfoung (B) and older adult (A)

We performed separation and identification of MHC isoforms following a previously described method^28^. Briefly, muscle blocks were cut into cryosections of 10 μm thickness in a cryostat (Thermo Electronic) cooled to −20°C. Myosin isoforms were separated using 6% sodium dodecyl sulfate polyacrylamide gel electrophoresis (SDS-PAGE) on a Bio-Rad Mini-PROTEAN gel system (Bio- Rad, Hercules, CA, USA). Electrophoresis was performed at 140 V for 10 h. The gels were subsequently stained with Bio-Safe Coomassie blue (Bio-Rad, Hercules, CA, USA). In the MHC isoform region, three major bands were separated in order of migration to MHC I or IIa and IIx depending on their molecular masses compared with those of marker proteins. Each MHC isoform was expressed as percentage of total densitometry. Densitometry was performed using ImageJ software (National Institutes of Health, MD, USA) (Figure 1).

### Statistical analysis

All data are presented as mean±SD. A two-way analysis of variance was used to determine the main effects of sex and age and age-by-sex group interaction on muscle mass, strength, and quality. Subsequent comparisons of age groups were performed using post-hoc analysis by independent t-test. Pearson’s correlation (r) coefficients were used to assess the relationship between MHC isoforms and muscle strength with respect to sex and age. All statistical analyses were performed using SPSS version 20.0 (IBM, Armonk, NY, USA), and p-values of <0.05 were considered statistically significant.

## RESULTS

### Anthropometric characteristics

The anthropometric characteristics of participants are summarized in Table 1. Older men were significantly smaller (*p*<0.001) and lighter (*p*=0.007) than young men. In contrast, older women were significantly smaller in height than young women (*p*=0.044), but no significant difference in weight was observed, with older women even showing a strong tendency.

### Body composition

Data on body composition including skeletal muscle mass (SMM), lean body mass (LBM), fat mass, and SMI according to age and sex are presented in Table 2. There were significant interactions between age-by-sex effects on SMM (*F*=19.553, *p*<0.001), LBM (*F*=38.763, *p*<0.001), percentage of body fat (*F*=7.817, *p*<0.001), and SMI (*F*=15.294, *p*<0.001). The SMM and LBM did not differ between young and older adults. However, these parameters were significantly greater in young men than in older men (SMM: 18.6%, *p*=0.026; LBM: 10.1%, *p*=0.014). In contrast, there were no significant differences in women according to age groups.

**Table 2.** The differences in body composition between younger and older adults

Characteristic	Y (n=28)	O (n=25)	YM (n=17)	OM (n=15)	YW (n=11)	OW (n=10)	Interaction (gender X age)
Skeletal muscle mass, kg	29.48±6.82	24.81±3.96	34.02±4.28	27.71±2.52a	23.8±4.71	21.18±1.69	19.553***
Lean body mass, kg	41.69±11.02	45.04±6.80	55.36±6.0	49.77±31.36a	35.48±5.65	37.95±38.47	38.763***
Fat mass, kg	17.51±6.25	16.88±6.33	15.46±4.53	15.62±4.84	18.44±6.88	18.77±7.98	0.793
% body fat, %	30.19±8.53	26.98±8.02	21.66±4.43	23.59±6.31	34.06±6.95	32.06±7.84	7.817***
SMI, kg/m^2^	0.07±0.01	0.07±0.01	8.08±0.94	7.97±0.71	5.96±1.12	6.37±0.79	15.294***

The data presented as mean±SD. Y, young adult; O, older adult; YM, young men; OM, older men; YW, young women; OW; older women. The skeletal muscle mass index (SMI) was defined as the ratio of appendicular skeletal muscle mass (ASM) by height squared. *P* values were calculated by independent T-Test. Significantly different between groups (a, YM vs. OM; b, YW vs. OW). Statistical significance was set at *p*<0.05.

### Muscle strength, function, and quality

We assessed grip strength, isometric/isokinetic strength, and isotonic power as objective measurements of muscle strength and quality. The results of sex-related differences in muscle strength and quality with aging are presented in Table 3. Except for lower-body muscle quality (*F*=2.843, *p*=0.051), which showed no significant interaction between age-by-sex effects, there were significant interactions between the effects of grip strength (*F*=18.751, *p*<0.001), isometric strength (*F*=12.446, *p*<0.001), isotonic power (*F*=33.423, *p*<0.001), isokinetic extensor (*F*=21.286, *p*<0.001) and flexor (*F*=10.395, *p*<0.001) strength, relative isokinetic extensor (*F*=24.35, *p*<0.001) and flexor (*F*=16.089, *p*<0.001) strength, and upper-body muscle quality (*F*=14.758, *p*<0.001). Older men showed significantly lower muscle strength, including grip strength (35.3%, *p*<0.001), isometric strength (29.6%, *p*=0.001), isotonic power (42.5%, *p*<0.001), isokinetic extensor (44.3%, p<0.001) and flexor (41.4%, *p*<0.001) strength, and relative isokinetic extensor (38%, *p*<0.001) and flexor (35.1%, *p*<0.001) strength, than young men. The muscle quality was poorer in older men than in young men (upper-body: 18.2%, *p*=0.022; lower-body: 24.8%, p=0.005). In contrast, older women had significantly lower values for isometric strength (24.2%, *p*=0.034), isotonic power (36.5%, *p*=0.002), relative isokinetic flexor strength (24.5%, *p*=0.01), and upper-body muscle quality (24.7%, *p*<0.001) only than young women, and other parameters of muscle strength and quality were not significantly different between age groups.

**Table 3.** The differences in muscle strength, function and quality between younger and older adults

Characteristic	Y (n=28)	O (n=25)	YM (n=17)	OM (n=15)	YW (n=11)	OW (n=10)	Interaction (gender X age)
Grip strength, kg	41.92±14.09	29.43±7.13*	51.35±8.84	33.21±6.32a	27.34±5.06	23.76±3.73	18.751***
Isometric strength, kg	168.83±50.24	136.13±43.83*	221.9±9.39	156.16±40.75a	139.88±37.18	106.09±29.49b	12.446***
Isotonic power, W	104.00±42.90	72.33±26.57*	152.62±25.78	87.76±20.8a	77.48±20.5	49.18±14.74b	33.423***
Isokinetic extensor strength, Nm	137.65±53.18	87.75±21.70*	173.84±31.57	96.79±17.0a	81.72±19.32	74.19±21.59	21.286***
Relative isokinetic extensor strength, Nm/kg (%)	197.88±60.45	136.85±32.25*	232.62±50.1	144.17±31.46a	144.19±25.04	125.87±31.78	24.350***
Isokinetic flexor strength, Nm	69.67±25.52	44.41±15.80*	86.3±17.32	50.56±16.03a	43.97±9.06	35.18±10.39	10.395***
Relative isokinetic flexor strength, Nm/kg (%)	101.15±29.18	68.79±22.85*	115.72±26.41	75.07±25.64a	78.65±16.3	59.36±14.33b	16.089***
Upper-body muscle quality, kg/kg	15.49±2.32	11.60±2.09*	13.61±2.07	11.13±1.87a	16.35±1.95	12.31±2.3b	14.758***
Lower-body muscle quality, Nm/kg	14.38±2.79	12.33±2.78*	16.09±3.23	12.1±2.18a	13.61±2.32	12.67±3.62	2.843

The data presented as mean±SD. Y, young adult; O, older adult; YM, young men; OM, older men; YW, young women; OW; older women. Muscle quality was defined as the ratio of strength by lean mass. *P* values were calculated by independent T-Test. Significantly different between groups (a, YM vs. OM; b, YW vs. OW). Statistical significance was set at *p*<0.05.

### Myosin heavy chain isoforms

**Fig. 2.**
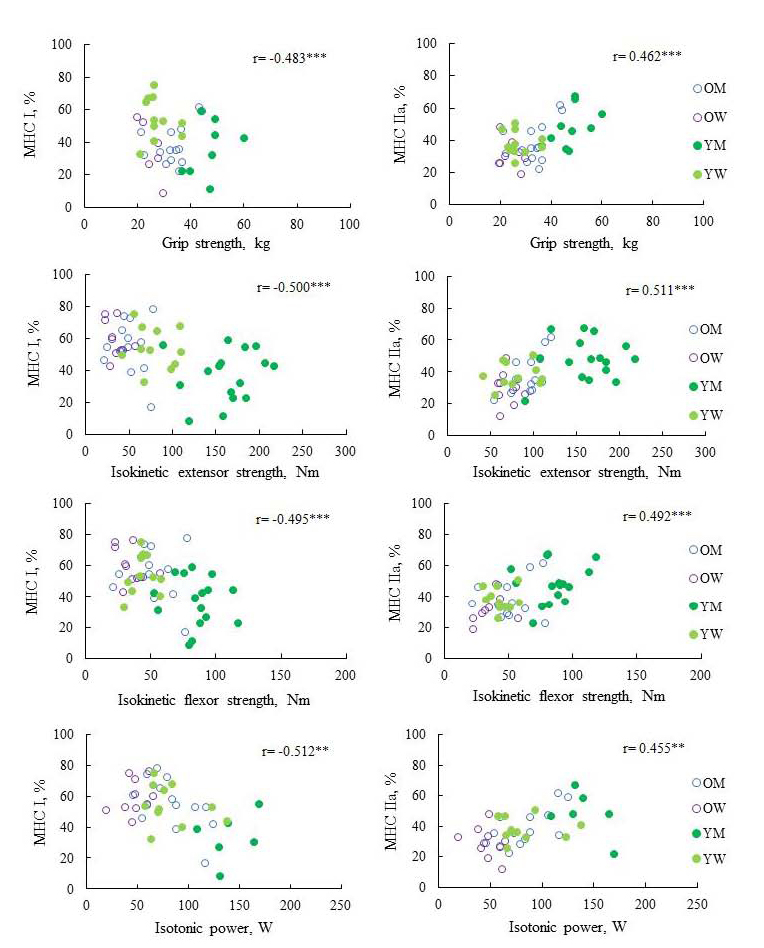
The association of myosin heavy chain isoform composition withm uscle strength and function (A) Association between MHC I and muscle strength, muscle function (B ) Association between MHC IIa and muscle strength, muscle functino YM, young men; OM, older men; YW, young women; OW; older women. Pearson’s correlation coefficients are shown and statistical significant was set at p<0.05.

The results for the MHC isoforms from the vastus lateralis and the correlations of these isoforms with muscle strength and quality are presented in Tables 4 and 5 and Figure 2. The proportion of MHC I was significantly higher in older adults than in young adults (*p*=0.005), whereas the proportion of MHC IIa was significantly lower in older adults than in young adults (*p*=0.008). Moreover, the proportion of MHC IIx appeared lower in older adults than in young adults, albeit without significant difference. Although the proportion of MHC IIx (*F*=2.329, *p*=0.087) did not show significant interactions between age-by-sex effects, there were significant interactions between the effects of MHC I (*F*=6.677, *p*=0.001) and MHC IIa (*F*=5.965, *p*=0.002). The proportion of MHC I was significantly higher in older men than in young men (*p*=0.005); however, there were no significant differences in the case of women. The proportion of MHC IIa was significantly lower in older men and women than in young men and women (*p*=0.045 and *p*=0.04, respectively), whereas significant differences in MHC IIx between the young and older groups were observed in men (*p*=0.033), but not in women. In addition, in all groups except for young men, the proportion of MHC I was found to be 42.5–103.9% higher than that of MHC IIa, whereas the proportion of MHC IIa was 29.8% higher than that of MHC I in young men. As shown in Table 5 and Figure 2, there was a negative correlation between MHC I and SMM (*r*=- 0.487, *p*<0.05), grip strength (*r*=-0.483, *p*<0.001), isometric strength (*r*=-0.593, *p*<0.001), isotonic power (*r*=- 0.512, *p*=0.001), isokinetic strength (extensor: *r*=-0.500, *p*<0.001; flexor: *r*=-0.495, *p*<0.001), and relative isokinetic strength (extensor: *r*=-0.459, *p*=0.001; flexor: *r*=-0.448, *p*=0.001). In contrast, MHC IIa positively correlated with SMM (*r*=0.521, *p*<0.01), grip strength (*r*=0.462, *p*=0.001), isometric strength (*r*=0.607, *p*<0.001), isotonic power (*r*=0.455, *p*=0.003), isokinetic strength (extensor: *r*=0.511, *p*<0.001; flexor: *r*=0.492, *p*<0.001), relative isokinetic strength (extensor: *r*=0.524, *p*<0.001; flexor: *r*=0.508, *p*<0.001), and lower-body muscle quality (*r*=0.358, *p*=0.023). There was no significant association between MHC IIx and muscle mass, strength, function, and quality.

**Table 4.** The differences in myosin heavy chain isoforms between younger and older adults

MHC isoforms	Y (n=28)	O (n=25)	YM (n=17)	OM (n=15)	YW (n=11)	OW (n=10)	Interaction (gender X age)
MHC-I	43.84±16.84	56.56±14.18*	36.72±15.74	54.44±16.04a	54.21±12.9	59.53±11.19	6.677**
MHC-IIa	43.66±11.80	34.45±11.85*	47.68±12.73	38.21±11.97a	37.82±7.45	29.19±9.95b	5.965**
MHC-IIx	12.50±10.22	9.00±9.27	15.62±10.56	7.36±9.61a	7.97±8.11	11.3±8.73	2.329

The data presented as mean±SD. Y, young adult; O, older adult; YM, young men; OM, older men; YW, young women; OW; older women. P values were calculated by independent T-Test. Significantly different between groups (a, YM vs. OM; b, YW vs. OW). Statistical significance was set at *p*<0.05.

**Table 5.** The association of myosin heavy chain isoform composition with muscle strength and function

MHC isoforms	IIx	IIa	I
Age	-0.206	-0.369**	0.399**
Skeletal muscle mass	0.188	0.521**	-0.487*
Total fat mass	0.287	-0.289	0.042
SMI	0.242	0.163	-0.262
Grip strength	0.231	0.462**	-0.483***
Isometric strength	0.226	0.607***	-0.593***
Isotonic power	0.282	0.455**	-0.512**
Isokinetic extensor strength	0.199	0.511***	-0.500***
Isokinetic flexor strength	0.215	0.492***	-0.495***
Relative isokinetic extensor strength	0.110	0.524***	-0.459**
Relative isokinetic flexor strength	0.114	0.508***	-0.448**
Upper-body muscle quality	-0.201	0.042	0.088
Lower-body muscle quality	0.063	0.358*	-0.299

Pearson’s correlation coefficients are shown and statistical significant was set at *p*<0.05.

## DISCUSSION

In the present study, we found sex-related differences in body composition (SMM, LBM), muscle strength (grip strength, isokinetic extensor and flexor strength, isometric strength, isotonic power), muscle quality, and MHC isoforms with aging in Korean men and women. During the aging process, age plays a major role in the correlation between MHC isoforms and body composition and muscle strength and quality in men, but it does not seem to have a significant effect on several measures of muscle parameters in women. These differences seem to occur because muscle strength and quality in men decrease with aging, with young and older women showing no difference. In particular, grip strength (27.34±5.06 vs. 23.76±3.73), isokinetic extensor (81.72±19.32 vs. 74.19±21.59) and flexor (43.97±9.06 vs. 35.18±10.39) strength, and lower-body muscle quality (13.61±2.32 vs. 12.67±3.62) were not significantly greater in young women than in older women. With respect to MHC isoform distributions, the proportion of MHC IIa and IIx and that of MHC I were significantly higher and lower, respectively, in young men than in older men. Only the proportion of MHC IIa was lower in older women than in young women, with no difference in the proportion of MHC I and IIx between the two groups.

Numerous studies have suggested that the decrease in SMM and muscle strength due to aging was characterized by a decline in the number of muscle fibers and, in particular, the atrophy of MHC IIa fibers^18-20, 29-31^. Landi et al.^32^ reported that older adults aged >75 years lose approximately 60% of their muscle strength. Further, they showed a similar linear pattern of age decline in both men and women across their entire life course. Interestingly, our study showed that these reductions differed by sex. In women, there were no significant differences in SMM, grip strength, and isokinetic knee strength between the young and older groups. Similarly, previous studies reported that the reduction in muscle quality^10^ and strength with weight loss was more pronounced in men than in women^5,33^. In the Survey of National Physical Fitness^16^, the grip strength was 44.5 kg in Korean men in their 20s (25–29 years of age) and 34.4 kg in men in their 70s (70–75 years of age), which was a difference of approximately 22.7%. In contrast, there was no significant difference in the grip strength between young and older women, with a difference of 13.1% (26 kg vs. 22.6 kg). Kim et al.^15^ also showed that the muscle mass and SMI were not significantly higher in Korean young women, indicating that the age-related difference was not significant in Korean women. In fact, the decline in muscle function including muscle strength attenuation is well known to be more remarkable in men than in women. However, we could explain the possible reasons for this sex-related difference based on the muscle parameter findings in the present study. The level of muscle mass and strength in Korean young women is not considerably different from that in older women, compared with the differences in men. When it comes to analyses of data on MHC isoforms from the vastus lateralis, the proportion of MHC IIa fibers in young men was 29.8% higher than that of MHC I fibers, whereas the proportion of MHC I fibers in older men was 42.5% higher than that of MHC IIa fibers, suggesting the dominant reduction in fast-twitch type II fibers due to aging. Surprisingly, MHC IIa fibers were 30.2% less in Korean young women than MHC I fibers. We consider that low muscle strength and quality in Korean young women may be associated with low proportion of MHC IIa fibers. Interestingly, we showed that the proportion of MHC IIx in men significantly decreased with aging, whereas those in women tended to increase. The MHC IIx isoform increases in pathological conditions such as cerebral palsy^34^ and spastic paresis^35^ and even in disuse-induced muscle wasting^36,37^, and it overshoots during detraining after the heavy-load resistance exercise training period^38^. However, in this study, the standard deviation for MHC IIx isoform might be too large to be associated with muscle strength and quality, unlike MHC IIa and I isoforms. MHC IIx isoform was not expressed in 18 of 53 participants (34%) in our study, who only had MHC I and IIa isoforms. In fact, MHC IIx is not often expressed in the human skeletal muscle.

This study has potential methodological limitations. Firstly, this is a cross-sectional study, and longitudinal studies are ideally required to be performed to validate our results. Secondly, our study has a limited sample size; hence, future studies with larger sample size and increased sampling areas are needed to confirm our findings. Finally, although muscle weakness due to aging is caused by various problems, we did not investigate metabolism, physical activities and lifestyle, living environment, and so on. In particular, this study did not investigate nutritional intake, which has an important effect on muscle function with aging. Nevertheless, this study is the first to report sex-related differences in MHC isoform compositions and muscle strength and quality with aging in the Korean population.

In conclusion, the results of this study indicate the existence of sex-related differences in muscle mass, strength, and quality with increasing age. The effects on muscle strength and quality with aging were significant in men, but not in women. Higher and lower proportions of MHC I and MHC IIa fibers, respectively, were inversely associated with muscle strength and quality. In particular, Korean young women showed lower muscle strength and quality, and the proportion of MHC isoforms was similar to that in the muscles of older women.
